# Surgical Management for Transposed Ovarian Recurrence of Cervical Cancer: A Systematic Review with Our Experience

**DOI:** 10.3390/curroncol29100563

**Published:** 2022-09-29

**Authors:** Michihide Maeda, Tsuyoshi Hisa, Hiroki Kurahashi, Harue Hayashida, Misooja Lee, Reisa Kakubari, Shinya Matsuzaki, Seiji Mabuchi, Shoji Kamiura

**Affiliations:** 1 Department of Gynecology, Osaka International Cancer Institute, Osaka 541-8567, Japan; 2 Department of Forensic Medicine, School of Medicine, Kindai University, Osaka 589-8511, Japan

**Keywords:** cervical cancer, ovarian metastasis, ovarian transposition, laparoscopic resection

## Abstract

In early-stage cervical cancer, ovarian metastasis is relatively rare, and ovarian transposition is often performed during surgery. Although rare, the diagnosis and surgical approach for recurrence at transposed ovaries are challenging. This study focused on the diagnosis and surgical management of transposed ovarian recurrence in cervical cancer patients. A 45-year-old premenopausal woman underwent radical hysterectomy, bilateral salpingectomy, and pelvic lymphadenectomy following postoperative concurrent chemoradiotherapy for stage IB1 cervical cancer. During the initial surgery, the ovary was transposed to the paracolic gutter, and no postoperative complications were observed. Ovarian recurrence was diagnosed using positron emission tomography–computed tomography, and a laparoscopic bilateral oophorectomy was performed. A systematic review identified nine women with transposed ovarian recurrence with no other metastases of cervical cancer, and no studies have discussed the optimal surveillance of transposed ovaries. Of those (n = 9), four women had died of the disease within 2 years of the second surgery, and the prognosis of transposed ovarian cervical cancer seemed poor. Nevertheless, three women underwent laparoscopic oophorectomies, none of whom experienced recurrence after the second surgery. Few studies have examined the surgical management of transposed ovarian recurrence. The optimal surgical approach for transposed ovarian recurrence of cervical cancer requires further investigation.

## 1. Introduction

Cervical cancer is the fourth most common malignant disease in women globally, with approximately 600,000 new cases and 340,000 deaths annually [1]. Notably, cervical cancer is most frequently diagnosed in women between the ages of 35 and 44 years, and the prognosis for women with cervical cancer mainly depends on the stage [2]. Surgery is often the primary treatment for cervical cancer in women with early-stage disease [2,3]. The incidence of ovarian metastasis in the early stage of squamous cell cervical cancer is 0.22–2.17%, while that in the early stage of cervical adenocarcinoma is 3.72–9.85% [4]. 

Ovarian preservation and transposition are considered feasible in women who opt for them, considering the low rate of ovarian metastasis from cervical cancer with squamous cell carcinoma (SCC) [1,5]. Conversely, ovarian preservation in cervical adenocarcinoma patients is more controversial as several studies have reported a high rate of ovarian metastasis with wide range (1.0–12.9%) [6,7,8,9,10,11,12,13,14]. A systematic review reported that the rate of ovarian metastasis was approximately 2% in women with stage IB cervical adenocarcinoma [15]. Therefore, further studies that examine the rate of ovarian metastasis in women with cervical adenocarcinoma are warranted to provide insight into the need for ovarian transposition.

Pelvic radiotherapy or concurrent chemoradiation invariably results in ovarian failure in women with ovarian preservation [16,17]. Therefore, ovarian transposition may be considered during surgery for early-stage cervical cancer to preserve intrinsic hormonal function [1]. A recent systematic review showed that ovarian function was preserved after ovarian transposition in approximately 60% (16.6% to 100%) of women with cervical cancer after radiotherapy [18,19].

Although the rate of ovarian metastasis is rare, ovarian transposition needs to be performed for selected patients owing to concerns regarding ovarian metastasis. The optimal management of ovarian metastasis after ovarian transposition in women with cervical cancer is understudied, including the feasibility of laparoscopic resection versus laparotomy. The Laparoscopic Approach to Cervical Cancer Trial for primarily treating early-stage cervical cancer, a prospective phase III randomized controlled trial, reported that minimally invasive surgery is correlated with worse disease-free and overall survival than open surgery [20,21]. Nevertheless, studies examining the safety and feasibility of laparoscopic resection for recurrent cervical cancer or ovarian metastasis are scanty [22,23].

In this study, we performed a systematic review to evaluate the surgical approaches and outcomes in women with ovarian metastasis after transposition during surgery for cervical cancer. In addition, we describe a patient with ovarian metastasis of cervical cancer who underwent ovarian transposition during the initial surgery. The patient was successfully treated with the laparoscopic resection of recurrent ovarian metastasis following adjuvant chemotherapy, and no recurrence was observed five years after the second surgery.

## 2. Detailed Case Description

### 2.1. Preoperative Assessment

A 45-year-old premenopausal woman (gravida 2, para 2) was referred to our institution for early-stage cervical cancer treatment. According to the preoperative assessment, the patient was diagnosed with stage IB1 cervical cancer according to the 2009 International Federation of Gynecology and Obstetrics (FIGO). Since no signs of ovarian metastasis were observed, the patient opted for ovarian preservation. Subsequently, she underwent a radical hysterectomy, bilateral salpingotomy, pelvic lymphadenectomy, and ovarian transposition, and the ovary was placed in the paracolic gutter.

**Figure 1:**
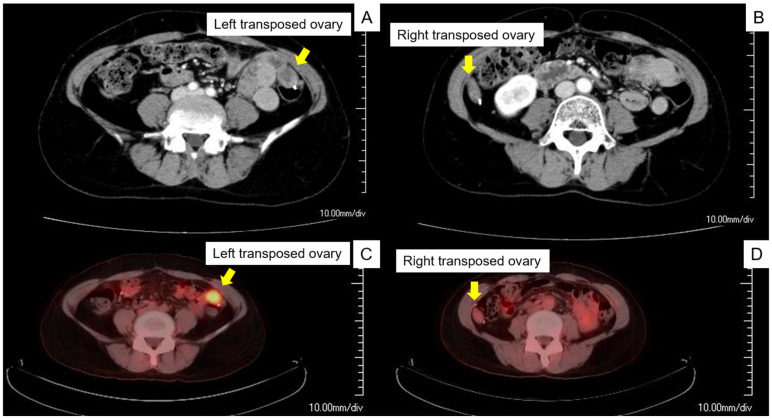
Clinical imaging of the recurrence of the transposed ovaries. (**A**) Contrast computed tomography (CT) scan revealed a slightly swollen left transposed ovary located in the uterine corpus. (**B**) The right ovary was not swollen on the contrast CT scan. (**C**,**D**) PET-CT revealed increased 18F-fluorodeoxyglucose uptake in the left transposed ovary and no increase in the right ovary. Yellow arrows indicate the left and right ovaries, respectively. Abbreviation: PET-CT, positron emission tomography–computed tomography.

Postoperative pathological findings identified parametrium invasion; therefore, concurrent chemoradiotherapy was administered at 50.4 Gy in 28 fractions and four cycles of cisplatin (40 mg/m^2^) weekly following the National Comprehensive Cancer Network guidelines. Cervical cytology, serum SCC antigen, and transvaginal ultrasonography were performed as postoperative surveillance every 3 months, and contrast computed tomography (CT) was performed twice a year. Three years after initial treatment, the serum SCC antigen level increased (3.1 ng/mL, reference ≤ 1.5 ng/mL), and contrast CT revealed swelling in the left transposed ovary, a sign of recurrence, whereas the right transposed ovary had no obvious abnormal findings (Figure 1A,B). Furthermore, positron emission tomography (PET)-CT was undertaken for further investigation. PET-CT showed an increased maximum standard uptake value of 4.8 in the left transposed ovary, whereas no increased 18F-fluorodeoxyglucose uptake in the right transposed ovary was observed (Figure 1C,D). We diagnosed the patient with an isolated ovarian recurrence, and laparoscopic bilateral oophorectomy was planned to treat the ovarian recurrence. 

### 2.2. Intraoperative Findings

**Figure 2:**
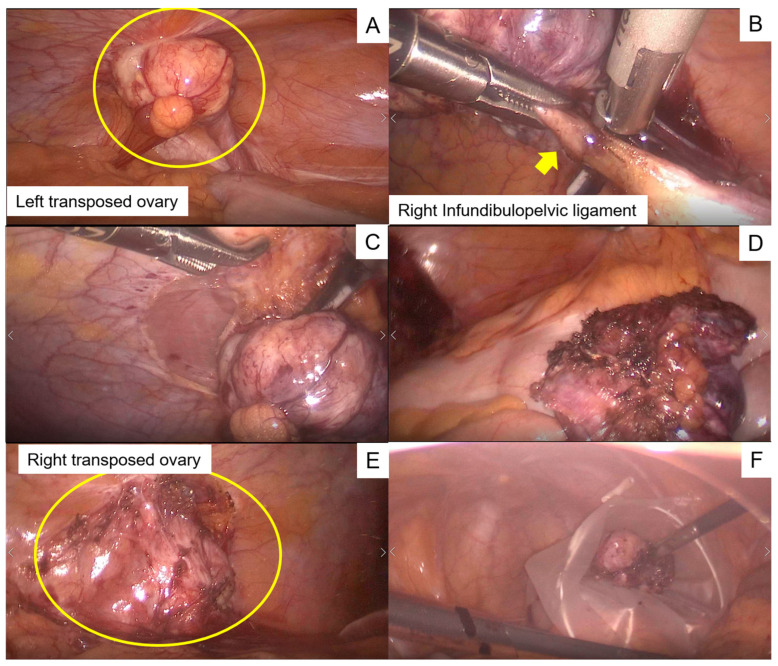
Intraoperative images. (**A**) A 3 cm tumor was observed in the paracolic gutter (yellow circle) and connected to the infundibulopelvic ligament. (**B**) The infundibulopelvic ligament (yellow arrow) was isolated and ligated. (**C**) The peritoneum around the tumor was incised. (**D**) The ovary was removed with the peritoneum. (**E**) The right ovary was identified after lysing the omentum (yellow circle) (**F**) The right ovary was removed, similar to the left ovary. The resected ovaries were placed in a plastic bag.

Laparoscopic bilateral oophorectomy was performed. During surgery, the adhesion between the omentum and the pelvic peritoneum was initially removed. Peritoneal washout cytology was negative. A 3 cm sized tumor was observed in the left paracolic gutter and was attached to the infundibulopelvic ligament (Figure 2A). Next, the infundibulopelvic ligament was isolated and ligated (Figure 2B). Subsequently, we incised the peritoneum around the tumor and removed the ovary with the peritoneum (Figure 2C,D). The right ovary could not be identified because of omentum adhesion. Subsequently, we lysed the adhesion of the omentum with the peritoneum, identified the ovary, and removed it following a procedure similar to that used for the left ovary (Figure 2E,F). After placing the resected ovaries into a plastic bag, the specimens were extracted via the umbilicus, and the abdominal cavity was flushed with a large volume of saline.

### 2.3. Postoperative Course

The postoperative course was uneventful, and the patient was discharged on the fifth postoperative day. A histopathological analysis was performed, and the tumor was diagnosed as a metastatic ovarian tumor from cervical squamous carcinoma metastasis. Therefore, we considered that the recurrence risk was relatively high because the ovarian metastasis was hematogenous, and three cycles of paclitaxel (175 mg/m^2^) and carboplatin (AUC = 5) were administered triweekly for adjuvant chemotherapy. The patient had no recurrence for five years after the second surgery.

## 3. Systematic Review

A systematic review was conducted to evaluate the surgical approaches and outcomes in women with ovarian metastasis after transposition during surgery for cervical cancer. According to the 2020 edition of the Preferred Reporting Items for Systematic Reviews and Meta-Analyses statement [24], we conducted a systematic literature search in PubMed from its inception to 30 June 2022, as previously performed with slight modifications [25,26,27,28]. The keywords related to “cervical cancer” AND “ovarian transposition” or “transposed ovary” and “ovarian metastasis” or “recurrence”, and MeSH keywords for “cervical cancer” (Uterine Cervical Neoplasms) were used for the search (Supplemental File S1). 

The search was limited to English literature, and only studies involving the resection of ovarian metastasis for cervical cancer after ovarian transposition were included in the review. Two review authors (M.M. and Sh.M.) identified relevant studies by screening titles and abstracts. Many articles were excluded during title screening because they did not meet the requirements for study type and surgery for uterine cervical cancer. This systematic review was not pre-registered.

**Figure 3:**
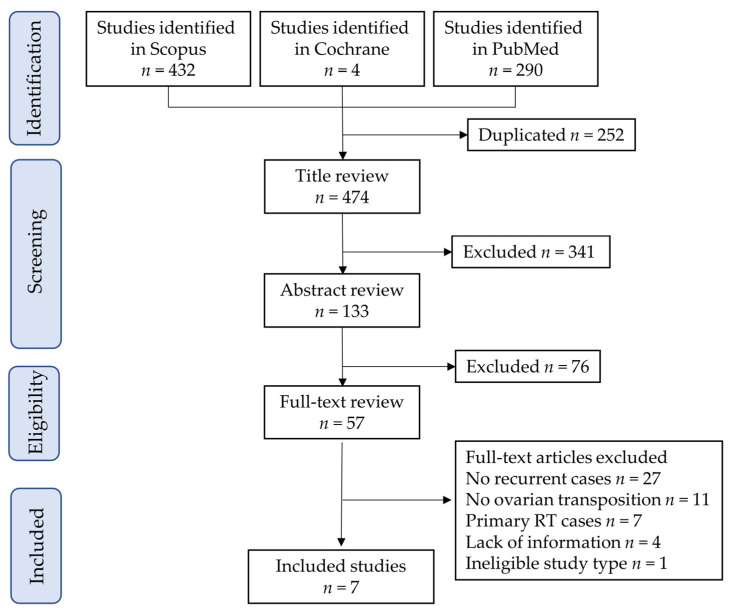
Study selection scheme of the systematic literature search. Abbreviation: RT, radiotherapy.

The inclusion criteria were as follows: (1) cervical cancer treated with surgical resection, including hysterectomy or trachelectomy; (2) studies that described a woman or women who had transposed ovarian recurrence; (3) no other metastasis except for ovary was identified; and (4) studies that discussed the optimal surveillance of recurrence in transposed ovaries. The exclusion criteria were as follows: (1) studies discussing ovarian transposition before primary radiotherapy; (2) a lack of information regarding ovarian transposition; and (3) conference papers, review articles, and systematic reviews. Based on these criteria, seven studies with eight eligible cases [29,30,31,32,33,34,35] were identified. The study selection scheme is illustrated in Figure 3, and the information, including our case, is summarized in Table 1. No study has discussed the optimal surveillance of recurrence in transposed ovaries.

Among the eligible women (*n* = 9), all patients other than ours had stage IB disease according to the International FIGO 2018 classification; four women had SCC, two had adenocarcinoma, two had adenosquamous carcinoma, and one had glassy cell carcinoma. The median age at recurrence was 36 (range, 31–53 years), and the median ovarian tumor size was 6 cm (3–10 cm). Among those (*n* = 9), no extra-ovarian recurrence was observed in none of the women.

The results of the systematic review suggest that our case study diagnosed an ovarian metastasis of relatively small size. In our case, we suspected the recurrence of cervical cancer due to the increased serum SCC antigen level, and ovarian recurrence was identified via 18F-fluorodeoxyglucose uptake on PET-CT. Therefore, transposed ovaries should be recognized as a possible recurrence site. After ovarian transposition, the location of the ovary changes, and CT might be needed to evaluate for features of ovarian recurrence. This background may lead to the detection of ovarian metastases, which are difficult to identify until they reach a larger size. 

The median time from the first treatment to recurrence was 36 months (range, 1–120 months). For six of the nine cases, the chief complaint was abdominal pain, while the others had no symptoms and were diagnosed at regular follow-up. Of those (*n* = 9), four recurrences were diagnosed by means of a CT scan; two by palpation; and one each by magnetic resonance imaging, ultrasonography, and PET-CT. With regard to the surgical approaches for transposed ovarian recurrence (*n* = 9), three were laparoscopic, three were open approaches, and data on the remaining three were unavailable. Six patients underwent postoperative adjuvant therapy; among these cases, three received radiotherapy, two received chemotherapy, and one received chemoradiotherapy. The median progression-free survival after the resection of transposed ovarian recurrence was 12 months (*n* = 7:0–60 months), and the median overall survival was 16.5 months (*n* = 8:5–60 months).

**Figure 4:**
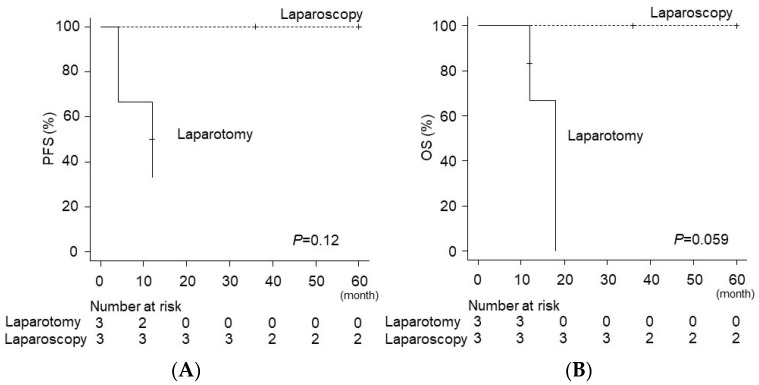
Survival curve estimates based on surgical approaches. Progression-free survival (PFS) and overall survival (OS) are shown based on surgical approaches. Cox proportional hazard regression model for *P*-value was performed as in our previous method, but with some modification [36]. Censored subjects are indicated on the Kaplan-Meier curve as + marks. (**A**) PFS for ovarian recurrent cases and (**B**) OS for ovarian recurrent cases.

Three of the nine cases had recurrence after the initial resection; one patient′s metastatic lesion persisted despite resection, and three patients died of the disease. After 2009, all the three patients with recurrence underwent laparoscopic resection with no further postoperative recurrence and achieved relatively long overall survival (Figure 4). A comparison of the progression-free survival and overall survival between the laparotomy (*n* = 3) and laparoscopic resection groups (*n* = 3) showed that the survival outcomes were similar between the groups (Figure 4). Therefore, we believe that the laparoscopic resection of ovarian metastases after transposition is safe and feasible.

## 4. Discussion

### 4.1. Principal Findings

The principal findings of this study are as follows: (*i*) ovarian recurrence after transposition is rare but should be recognized as a possible recurrence site; (*ii*) ovarian recurrence after transposition was successfully resected using the laparoscopic approach, achieving long-term recurrence-free survival; and (*iii*) even if PET-CT shows no bilateral abnormality, bilateral ovarian recurrence needs to be considered when unilateral ovarian metastasis is detected. 

### 4.2. Effect of Ovarian Metastasis in Cervical Cancer

Although ovarian metastasis is not frequent and is not classified by the FIGO staging system [37], some gynecologic oncologists think that ovarian metastasis has poor oncologic outcomes due to the increased risk of intraperitoneal recurrence [38]. A previous report showed poor oncologic outcomes in cervical cancer with ovarian metastasis; the 5-year survival rates were 46.6%, 37.5%, and 18.0% for stages IB, IIA, and IIB, respectively [4]. Ovarian recurrence is less common, and its prognosis is not well known. Given the possible poor prognosis of ovarian metastasis, we suggest that ovarian transposition should be performed in carefully selected women with cervical cancer.

### 4.3. Ovarian Transposition for Women with Cervical Cancer

Ovarian transposition was first reported in the 1970s for Hodgkin’s lymphoma and was performed before radiation therapy for various diseases, such as cervical cancer, vaginal cancer, uterine cancer, ovarian dysgerminomas, osteosarcoma, rhabdomyosarcoma, and anorectal cancer [39,40,41]. A previous systematic review showed that the rates of successful ovarian function preservation were 61.7%, 85.7%, and 51.1% after ovarian transposition, followed by radiotherapy with or without chemotherapy, brachytherapy, and chemoradiation, respectively. Moreover, the rate of ovarian metastasis after transposition was 0.36% [18]. Notably, the complication rate of ovarian transposition was <10% [18]. Therefore, ovarian transposition during surgery for cervical cancer is tolerable for women who wish to preserve ovarian function.

Ovarian transposition can be considered for women with early-stage cervical cancer who are premenopausal, with acceptable oncological risk of ovarian metastasis, and who are potential candidates for adjuvant radiotherapy [19]. According to the National Comprehensive Cancer Network (NCCN) guideline, ovarian transposition can be considered before pelvic radiotherapy in select young patients (<45 years old with early-stage disease) [42]. It is essential to balance the oncological risk of ovarian metastasis and the benefit of maintaining ovarian hormones [14]. Therefore, appropriate case selection for ovarian preservation needs to be discussed.

### 4.4. Ovarian Preservation for Women with Cervical Cancer

Ovarian preservation is a relevant problem for premenopausal women with cervical cancer. The benefits of gonadal hormones for women’s health are well studied [43,44,45,46]. A lack of ovarian hormones due to surgical resection can lead to menopausal symptoms, vaginal dryness, an increased rate of osteoporosis, and a high risk of cardiovascular morbidity and mortality [47,48,49]. Ovarian preservation should be determined on an individual basis, taking into account the patient′s individual oncologic risk and background. For instance, there is a complexity of the decision for ovarian preservation in women with Lynch syndrome or BRCA mutations, due to the high prevalence of ovarian cancer [50,51,52,53,54,55].

A recent retrospective, multi-center, observational cohort study examined the rate of ovarian metastasis/recurrence and the survival of women undergoing radical hysterectomy with and without oophorectomy. The study included 419 women with clinical FIGO 2009 stage IA1-IB1/IIA1 cervical cancer, with 264 and 155 women undergoing ovarian conservation and oophorectomy, respectively. In this study, a survival analysis after propensity-score matching was performed, and a significantly higher 5-year disease-free survival was observed in the ovarian conservation group than in the oophorectomy group (90.6% versus 82.2%, *P* = 0.028), whereas overall survival was similar between the groups (94.3% versus 90.8%, *P* = 0.157) [14]. Regarding the menopausal disorders, 28 women (20.6%) in the ovarian conservation group versus 116 (60.4%) in the oophorectomy group complained of menopausal symptoms (*P* < 0.01) [14]. 

In the study [14], the authors considered the following three merits of ovarian conservation if the oncologic risk was acceptable: (i) menopausal symptoms were less frequent in the ovarian conservation group than in the hormone replacement therapy with oophorectomy group; (ii) estrogen and progesterone have potential protective role in cervical carcinoma; and (iii) lower rates of metabolic syndrome, osteoporosis, cardiovascular events, and neurologic disorders.

The risk of ovarian metastasis in women with early cervical cancer (stages I-II) has been widely reported [6,7,8,9,10,11,12,13,14]. The rate of ovarian metastasis in SCC is low in early-stage cervical cancer (0–1.3%); thus, ovarian preservation in the case of SCC histology is feasible. In contrast, ovarian preservation in cervical adenocarcinoma patients is controversial, given the high heterogeneity in the rate of ovarian metastasis in previous studies [6,7,8,9,10,11,12,13,14]. The rate of ovarian metastasis in women with early cervical adenocarcinoma varies widely (1.0–12.9%), which explains the diverse opinions about ovarian preservation. 

A Japanese population-based, retrospective, observational study examined the risk of ovarian metastasis in early cervical cancer (clinical stages IB to IIB) and reported that cervical adenocarcinoma, uterine corpus invasion, lymph vascular space invasion (LVSI), and lymph-node metastasis (pelvic/para-aorta) were independent risk factors for ovarian metastasis [13]. Although cervical adenocarcinoma is a risk factor for ovarian metastasis, this study showed that the incidence of ovarian metastasis was 0.17% in women with cervical adenocarcinoma without the aforementioned risk factors. Therefore, in our opinion, ovarian preservation can potentially be performed in patients with cervical adenocarcinoma without LVSI, uterine corpus invasion, and no pelvic nor para-aortic lymph-node metastases.

### 4.5. Surgical Treatment for Metastatic Ovarian Tumors

Since the use of ovarian transposition is expected to increase, the number of ovarian metastases after ovarian transposition is expected to rise, requiring subsequent surgical resection. Metastatic ovarian tumors account for approximately 15% of all ovarian malignancies and originate from primary lesions at various sites, including the gastrointestinal tract, breasts, and endometrium [56]. The feasibility of laparoscopic resection for primary ovarian cancer and metastatic ovarian tumors remains debatable. Therefore, we consider laparotomy to be the standard surgical approach for ovarian metastatic malignant disease. Additionally, the laparoscopic approach was performed for selected cases, such as the resection of metastatic ovarian tumors, recurrent ovarian cancer with no widespread disease, and interval debulking surgery [23,29,57]. Notably, no randomized trials have been conducted for the laparoscopic resection of metastatic ovarian tumors; consequently, the oncologic outcome, safety, and feasibility are unknown.

For ovarian cancer, tumor capsule rupture worsens the oncologic outcomes and upgrades stages IA or IB to stage IC1 according to the 2014 International FIGO classification [58,59]. Therefore, to avoid surgical tumor spillage during resection, we incised the surrounding peritoneum without contacting the tumor; removed the ovary with the peritoneum; and extracted the specimen via the umbilicus using a flexible plastic bag. When the tumor was too large to be extracted via the umbilicus, in-bag morcellation or expansion of the surgical wound was performed to prevent intraperitoneal spill or port-site metastasis [60].

### 4.6. Identification of Recurrence of Cervical Cancer in Transposed Ovary

In this case, left ovarian metastasis was detected on preoperative imaging. The right ovary was not swollen and did not exhibit increased 18F-fluorodeoxyglucose uptake. However, postoperative pathological examination revealed bilateral ovarian metastasis. Approximately half of all ovarian metastases in cervical cancer are bilateral and microscopic; thus, bilateral oophorectomy is recommended when unilateral ovarian metastasis is detected [56,61].

Ovarian recurrence after transposition is difficult to diagnose using CT because of its anatomically changed position and the presence of small intestine around the transposed ovaries. In a previous report, ovarian metastasis was not diagnosed unless the metastatic tumor had reached a large size. While we used PET-CT imaging to diagnose recurrent disease, we should note that the ovary sometimes physiologically increases 18F-fluorodeoxyglucose uptake even after ovarian transposition; consequently, the diagnosis of transposed ovarian recurrence using PET-CT is challenging [62,63]. Therefore, increased 18F-fluorodeoxyglucose uptake should be interpreted with care.

### 4.7. Strengths and Limitations

The strength of the current case report and review is that they are likely the first to focus on the surgical approach and prognosis following resection after ovarian transposition. Notably, a previous systematic review of ovarian transposition during cervical cancer surgery identified three to six cases of ovarian recurrence [5,18,64]. Nevertheless, our systematic review identified nine cases, and these results demonstrated the robustness of our methodology. We believe that this study can be useful for clinicians to gain insights into the surgical approach for recurrence patterns after ovarian transposition. 

However, some salient limitations of this review and case report are acknowledged. First, our systematic review was based on case reports; therefore, publication bias may have influenced our findings. For instance, a poor prognosis of ovarian metastasis after transposition may not have been reported. Second, only a few similar cases were reported; therefore, this study as underpowered to discuss the feasibility of laparoscopic resection for ovarian metastasis after ovarian transposition. Therefore, future studies that report the feasibility of the laparoscopic resection of ovarian metastasis after transposition are warranted. 

Third, the ovarian tumor size was small in women with ovarian recurrence who were treated with laparoscopic resection. The feasibility of laparoscopic resection for large ovarian metastasis after ovarian transposition is still unknown. Fourth, the OS outcomes for two of the eight women with ovarian recurrence after ovarian transposition were unavailable in the systematic review. Our study included a limited number of women with ovarian recurrence after ovarian transposition; it was, thus, underpowered.

Fifth, we used three search engines to find all eligible cases, but more search engines might have enhanced the robustness of our literature search. However, due to the limited research resources of our institute, we could not use any more search engines. 

## 5. Conclusions

Herein, we describe a case of the successful laparoscopic resection of ovarian metastasis of cervical cancer after ovarian transposition. Some women with transposed ovarian recurrence were successfully treated using the laparoscopic approach, and the feasibility of the laparoscopic resection of ovarian metastasis after ovarian transposition is difficult to evaluate, owing to the limited number of studies. Further studies are warranted to prove the feasibility of laparoscopic resection for transposed ovarian recurrence.

## Figures and Tables

**Table 1:** Summary of identified studies for ovarian metastases after ovarian transposition.

Author	Year	Age	Stage ^†^	Hist	Rec	CC	Diagnosis	Surgery	Size	Adj	Rec	PFS	OS	Status
Present study	2022	45	IIB	SCC	36M	None	PET-CT	LSC	3 cm	CT	No	60M	60M	NED
Janse [30]	2011	53	IB	ASC	120M	Abd pain	US	LSC	6 cm	No	No	36M	36M	NED
Delotte [29]	2009	36	IB2	ADC	13M	Abd pain	MRI	LSC	4.5 cm	No	No	60M	60M	NED
Morice [34]	2001	34	IB	SCC	36M	Abd pain	Palp	-	10 cm	-	Yes	-	15M	DOD
Morice [34]	2001	34	IB	SCC	36M	None	Palp	-	10 cm	CCRT	-	-	-	-
Shigematsu [32]	2000	41	IB3	ASC	24M	None	CT	Open	7 cm	CT	No	12M	12M	NED
Nguyen [31]	1998	43	IB	SCC	98M	Abd pain	CT	Open	7 cm	RT	Yes	4M	18M	DOD
Parham [35]	1994	33	IB2	ADC	7M	Abd pain	CT	Open	6 cm	RT	Yes	12M	12M	DOD
Reisinger [33]	1991	31	IB3	Glassy	1M	Abd pain	CT	-	5 cm	RT	Yes	0 M	5M	DOD

^†^ Based on the International FIGO 2018 classification. Abbreviations: Hist, histology; SCC, squamous cell carcinoma; ASC, adenosquamous carcinoma; Glassy, glassy cell carcinoma; rec, time from initial treatment to recurrence; CC, chief complaint; Size, size of metastatic tumor; Adj, adjuvant therapy after oophorectomy for ovarian metastasis; CCRT, concurrent chemoradiotherapy; Palp, palpation; CT, chemotherapy; RT radiation therapy; Rec, recurrence; PFS, progression-free survival; NED, no evidence of disease; DOD, dead of disease; and OS, overall survival.

## Data Availability

The data presented in this study are available on request from the corresponding author.

## Supplementary Materials

The following supporting information can be downloaded at: https://www.mdpi.com/article/10.3390/curroncol29100563/s1, Supplemental File S1. Search keywords.
