# Supplementary material for: Surgical Management for Transposed Ovarian Recurrence of Cervical Cancer: A Systematic Review with Our Experience
Source: Curr Oncol. 2022 Sep 29;29(10):7158–70. doi: 10.3390/curroncol29100563 (PMC9600108; doi:10.3390/curroncol29100563)
Supplement: Supplementary file 1 [file curroncol-29-00563-s001.zip › curroncol-1893172-supplementary.pdf]

Supplemental File S1. Search keywords.

#1 Uterine Cervical Neoplasms [MeSH]
#2 "cervical neoplasm" OR "cervical cancer" OR "cervical carcinoma" OR "cervical malignancy"
#3 "uterine cervical neoplasm" OR "uterine cervical cancer" OR "uterine cervical carcinoma" OR "uterine cervical malignancy"
#4 "carcinoma of the cervix" OR "carcinoma of the uterine cervix"
#5 "squamous cell carcinoma of the cervix"
#6 "adenocarcinoma of the cervix" OR "adenocarcinoma of the uterine cervix"
#7 #1 OR #2 OR #3 OR #4 OR #5 OR #6
#8 "ovarian transposition" OR "transposed ovary" OR "transposed ovaries" OR "ovarian metastasis" OR "ovarian function" OR "ovarian management" OR "ovarian treatment"
#9 #7 AND #8

Scopus
#1 TITLE-ABS-KEY(cervical OR uterine cervix) W/2 (neoplasm OR cancer OR malignan* OR tumor OR tumour OR carcinoma)
#2 "uterine cervical neoplasm" OR "uterine cervical cancer" OR "uterine cervical carcinoma" OR "uterine cervical malignancy"
#3 "carcinoma of the cervix" OR "carcinoma of the uterine cervix"
#4 "squamous cell carcinoma of the cervix"
#5 "adenocarcinoma of the cervix" OR "adenocarcinoma of the uterine cervix"
#6 #1 OR #2 OR #3 OR #4 OR #5
#7 "ovarian transposition" OR "transposed ovary" OR "transposed ovaries" OR "ovarian metastasis" OR "ovarian function" OR "ovarian management" OR "ovarian treatment"
#8 #6 AND #7

Cochrane
#1 Uterine Cervical Neoplasms [MeSH]
#2 "ovarian transposition" OR "transposed ovary" OR "transposed ovaries" OR "ovarian metastasis" OR "ovarian function" OR "ovarian management" OR "ovarian treatment"
